# Predictive Dispatch of Volunteer First Responders: Algorithm Development and Validation

**DOI:** 10.2196/41551

**Published:** 2023-11-28

**Authors:** Michael Khalemsky, Anna Khalemsky, Stephen Lankenau, Janna Ataiants, Alexis Roth, Gabriela Marcu, David G Schwartz

**Affiliations:** 1 Department of Management Hadassah Academic College Jerusalem Israel; 2 School of Public Health Drexel University Philadelphia, PA United States; 3 School of Information University of Michigan Ann Arbor, MI United States; 4 The Graduate School of Business Administration Bar-Ilan University Ramat Gan Israel

**Keywords:** volunteer, emergency, dispatch, responder, smartphone, emergency response, smartphone-based apps, mobile phone apps, first responders, medical emergency, dispatch algorithms, dispatch decisions, dispatch prediction, smartphone app, decision-making, algorithm, mobile health, mHealth intervention, mobile phone

## Abstract

**Background:**

Smartphone-based emergency response apps are increasingly being used to identify and dispatch volunteer first responders (VFRs) to medical emergencies to provide faster first aid, which is associated with better prognoses. Volunteers’ availability and willingness to respond are uncertain, leading in recent studies to response rates of 17% to 47%. Dispatch algorithms that select volunteers based on their estimated time of arrival (ETA) without considering the likelihood of response may be suboptimal due to a large percentage of alerts *wasted* on VFRs with shorter ETA but a low likelihood of response, resulting in delays until a volunteer who will actually respond can be dispatched.

**Objective:**

This study aims to improve the decision-making process of human emergency medical services dispatchers and autonomous dispatch algorithms by presenting a novel approach for predicting whether a VFR will respond to or ignore a given alert.

**Methods:**

We developed and compared 4 analytical models to predict VFRs’ response behaviors based on emergency event characteristics, volunteers’ demographic data and previous experience, and condition-specific parameters. We tested these 4 models using 4 different algorithms applied on actual demographic and response data from a 12-month study of 112 VFRs who received 993 alerts to respond to 188 opioid overdose emergencies. Model 4 used an additional dynamically updated synthetic dichotomous variable, *frequent responder*, which reflects the responder’s previous behavior.

**Results:**

The highest accuracy (260/329, 79.1%) of prediction that a VFR will ignore an alert was achieved by 2 models that used events data, VFRs’ demographic data, and their previous response experience, with slightly better overall accuracy (248/329, 75.4%) for model 4, which used the *frequent responder* indicator. Another model that used events data and VFRs’ previous experience but did not use demographic data provided a high-accuracy prediction (277/329, 84.2%) of ignored alerts but a low-accuracy prediction (153/329, 46.5%) of responded alerts. The accuracy of the model that used events data only was unacceptably low. The J48 decision tree algorithm provided the best accuracy.

**Conclusions:**

VFR dispatch has evolved in the last decades, thanks to technological advances and a better understanding of VFR management. The dispatch of substitute responders is a common approach in VFR systems. Predicting the response behavior of candidate responders in advance of dispatch can allow any VFR system to choose the best possible response candidates based not only on ETA but also on the probability of actual response. The integration of the probability to respond into the dispatch algorithm constitutes a new generation of individual dispatch, making this one of the first studies to harness the power of predictive analytics for VFR dispatch. Our findings can help VFR network administrators in their continual efforts to improve the response times of their networks and to save lives.

## Introduction

### Background

Emergency response apps, commonly smartphone based, are increasingly being used to identify and dispatch volunteer first responders (VFRs) to the location of a medical emergency [1]. Automated dispatch algorithms generally rely on a simple estimated time of arrival (ETA) calculation based on the locations of the VFRs and the incident as well as the known modes of transport. A key aspect lacking in these algorithms is a consideration of the likelihood of response; for instance, given a set of potential VFRs with equivalent ETAs, which subset should be alerted to maximize the likelihood of response? The automated dispatch of VFRs to medical emergencies is suboptimal owing to a large percentage of alerts *wasted* on VFRs with shorter ETA but a low likelihood of response. This results in delays until a volunteer who will actually respond can be identified and dispatched. Using actual demographic and response data taken from a 12-month study of 112 VFRs alerted to respond to opioid overdose emergencies, we applied a series of analytical methods and advanced classification models to learn and predict volunteer response behaviors. Our findings can be used to improve dispatch algorithms in VFR networks to optimize dispatch decisions and increase the likelihood of timely emergency responses.

### Medical Emergencies

A medical emergency is an acute injury or illness that can result in death or long-term health complications [2]. Some common medical emergencies include out-of-hospital cardiac arrest (OHCA), severe trauma, opioid overdose, and anaphylaxis. OHCA is a leading cause of death worldwide [3], with a poor survival rate (only 5.6% in adults) [4]. Major trauma is the sixth leading cause of death worldwide [5,6]. Opioid overdose is a severe public health problem that has been consistently rising for the past 20 years and in the United States is the leading cause of accidental death [7]. The incidence of anaphylaxis ranges from 1.5 to 7.9 per 100,000 population per year in Europe [8,9].

### Networks of VFRs

The immediate provision of first aid is crucial in lowering mortality and improving long-term prognosis, particularly in regard to OHCA [10-12] and opioid overdose events [13]. Emergency medical services (EMS) are the primary first aid provider [14,15], but EMS response times vary significantly among countries and geographies [16,17]. Interventions to achieve faster response times include the deployment of automatic external defibrillators (AEDs) in public places [18-21] and the establishment of local networks of VFRs [22-30]. Recently, there was a concerted effort to use smartphone apps for faster emergency response, such as PulsePoint, HelpAround, Heartrunner, and UnityPhilly. An extensive review of emergency response apps can be found in the study by Gaziel-Yablowitz and Schwartz [31].

An emergency response community (ERC) [32], a subtype of a VFR network, is a social network of patients who are prescribed to carry life-saving medication for themselves and can potentially help other patients who are without their medication in a medical emergency. Two projects that apply the ERC approach are the subjects of recent field studies: EPIMADA, which focused on patients at risk of anaphylaxis and their parents [33]; and UnityPhilly, which focuses on people who have experienced an opioid overdose [34].

### Willingness to Respond, Barriers, and Facilitators

Once a person becomes a volunteer, they are expected to respond if available when a relevant event occurs. However, the actual rates of response to emergency alerts are far from 100%. Brooks et al [35] reported a response rate of 23% among PulsePoint volunteers. In a recent study, the willingness of cardiopulmonary resuscitation (CPR)–trained bystanders to respond to an OHCA event was 46.6% [36]. Another study analyzed barriers to receiving notifications and reported that 32% of the responders who were sent notifications did not receive the notification because, for example, they were away from their device (21%), their device was switched off (8%), or their device was out of network range (4%) [35]. Stress levels among responders varied for different medical conditions, different locations, and different demographic groups [37]. Younger age, higher education level, shorter time since the last CPR training, and cardiac arrest event in a public location were good predictors of bystanders’ greater willingness to perform CPR. The main reasons for not performing CPR were panic, the perception of bystanders that they are not able to perform CPR correctly, and a fear of hurting the patient [38]. Familial experiences of receiving CPR were associated with an increase in responders’ willingness to perform CPR [39]. The UnityPhilly study, which established a network of volunteers to provide naloxone to those experiencing an opioid overdose, reported that 17% of the alerted volunteers accepted the alert, and 11.9% of the alerted volunteers arrived at the scene [34].

### Dispatch Algorithms and Decision-Making

#### Complexity of VFR Dispatch and Decision-Making

The complexity of VFR dispatch stems from 2 sources: unknown resource location and uncertain response. Emergency response services that try to optimize their own resources to maximize their effectiveness can determine the allocation of their resources, such as ambulance dispatch stations or police patrol districts, subject to constraints (eg, budgets) [40-42]. The administrators of a VFR network are unable to plan and control the location of their resources because VFRs perform their daily activities until called to action: they can be anywhere, enter and exit the area that the network covers, switch on and off their mobile phones, and so on. In addition, although ambulance staff or a police patrol are expected to respond to any event that they are dispatched to, VFRs decide for themselves whether to respond to a specific event.

#### Usual Location–Based Dispatch Approach Using Pagers and SMS Text Messages

In a typical location-based approach, VFRs are alerted based on their usual location (eg, home or work address) and not their actual location at the moment of the alert. VFRs may not provide any feedback to the system regarding their availability to respond to the specific event and just show up on the scene if they can; for example, this approach was used by Zijlstra et al [43] who sent SMS text messages to volunteers living within a 1000-meter radius of an OHCA event.

#### Current Location–Based Dispatch Approach

A current location–based dispatch approach is based on a smartphone app that continuously sends VFRs’ locations (eg, geospatial coordinates) to a central server. When an emergency event is registered in the system, the dispatch algorithm selects volunteers based on their distance from the scene or, in a more advanced version, based on their ETA [44]. Such apps can also allow VFRs to set their availability status to control for their commitment, which was found to be an important factor of VFRs’ willingness to volunteer [45]. Location-based dispatch is widely used in VFR networks [34,36,46,47]. Usually, location-based algorithms dispatch >1 volunteer, if available, but still limit the number of volunteers who are dispatched to prevent burnout and a decrease in self-efficacy. Sending a large number of responders to each event can lead to the “diffusion of responsibility” phenomenon and reduce willingness to respond [48].

#### Autonomous Dispatch Versus EMS-Mediated Dispatch

Some VFR networks are managed by EMS and are integrated into their business processes. In this case, the dispatch of VFRs is at the discretion of a human dispatcher, and the VFR system serves as a decision support system that provides the dispatcher with the necessary information, such as location and ETA, of volunteers that can be compared with the location and ETA of an ambulance. Once alerts are sent, the system constantly updates its recommendations based on the feedback from the alerted volunteers. This approach is used by the Life Guardians project managed by Israeli National EMS [46] and in several AED and CPR projects [36].

An alternative approach is autonomous dispatch, where VFRs are selected and alerted by an autonomous system according to a predefined business logic. The system can dispatch additional volunteers if the alerted volunteers ignore the alert, refuse to respond, or linger on the way. This approach was used by the UnityPhilly project [34] and the PulsePoint project [35].

Both approaches can be either registered (usual or expected) location based or current (dynamic) location based; for example, UnityPhilly uses a current location–based autonomous dispatch approach.

#### Integration of Volunteers’ Feedback Into Dispatch Algorithms

**Figure 1:**
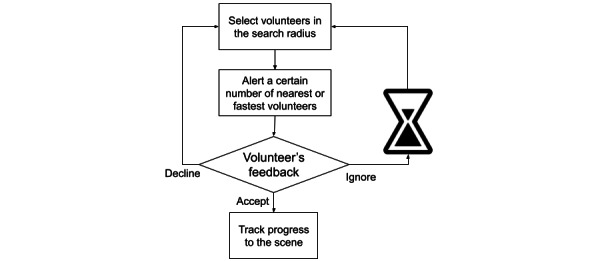
The dispatch process and feedback from alerted volunteers.

Many smartphone apps for VFR networks allow alerted responders to accept or decline the alert. Such feedback lowers the uncertainty regarding the dispatcher, and, if a volunteer declines an alert, the dispatch algorithm can reconsider the selection of responders and send additional alerts to substitute volunteers (ie, to volunteers who were not initially selected by the algorithm [eg, because they had a longer ETA] but who, in the event that ≥1 of the initially selected volunteers decline or ignore the alert, can be dispatched to achieve the target number of responders). If an alerted volunteer ignores the alert and does not provide any feedback, the system waits for a set period of time and then considers the nonresponse a “no” and acts accordingly. Figure 1 depicts this process.

### Profiling

Profiling is “the process of generating profiles from obtained data, associated to one or multiple subjects” [49]. Profiling of people is widely used in several areas, such as targeted advertising [50], donation solicitation [51], and volunteer recruitment [52]. Elsner et al [22,49] proposed to use the profiling of volunteers in dispatch algorithms to enhance the prediction of the volunteers’ position, trajectory, and constraints. In this study, we used classification techniques to generate different behavioral profiles of volunteers that serve as independent variables for predicting responses to alerts.

### The Purpose of the Study

The challenge of improving volunteer dispatch speed and response rates is recognized in fields ranging from food rescue operations [53] to OHCA response in which the optimization of the responder network is now taking center stage [54,55]. Studies such as the one by Gregers et al [56] have attempted to determine the optimal number of responders to dispatch, yet such studies base response viability solely on current ETA with no consideration of responder history or other characteristics that could improve responsiveness. Currently used dispatch algorithms that select volunteers based on their ETA without considering the likelihood of response may be suboptimal owing to a large percentage of alerts *wasted* on VFRs with shorter ETA but a low likelihood of response. We build on prior work on VFR optimization by presenting a novel approach for predicting whether a VFR will respond to, or ignore, a given alert. As such, the enhanced algorithm reduces the time that the system unnecessarily spends waiting for a response from volunteers who are likely to ignore the alert. The amount of time wasted depends on the specific dispatch algorithm; for example, in UnityPhilly trials, the system waited 2 minutes before dispatching a substitute volunteer. A faster dispatch of substitute volunteers has the potential to reduce the response time of the VFR network as a whole and improve its effectiveness. However, overdispatch of more VFRs than necessary to secure an effective emergency response can have a negative impact on future willingness to respond [48].

## Methods

### Data

We used data from the UnityPhilly study that piloted a smartphone-based app for requesting and providing ERC assistance to those suspected of experiencing an opioid overdose in the neighborhood of Kensington, PA, over 12 months from March 1, 2019, to February 28, 2020. Kensington has Philadelphia’s highest concentration of overdose deaths and is also home to Prevention Point Philadelphia, which is a city-sanctioned syringe exchange program that also distributes naloxone and provides naloxone training. Recruitment occurred via face-to-face screening at Prevention Point’s drop-in center, Prevention Point’s substance use disorder treatment van, street intercepts, and chain referrals from enrolled participants. The inclusion criteria for participants were that they lived, worked, or used drugs within 4 zip codes around the Kensington neighborhood (19122, 19125, 19133, and 19134); possessed a smartphone with a data plan; were willing to have location and movements tracked via an app; were willing to carry naloxone; and were aged ≥18 years. Sampling purposefully targeted a mix of members of the Kensington community who used opioids nonmedically in the past 30 days and those who reported no nonmedical opioid use in the past 30 days. The study recruited 112 volunteers who were almost equally divided between people who reported opioid use in the past 30 days at baseline (n=57, 50.9%) and community members, that is, people who reported no opioid use at baseline (n=55, 49.1%).

At a research storefront in Kensington, the study enrollment procedure included obtaining written informed consent, the recording of contact information, structured baseline interviews, app installation and training, and naloxone distribution and training. During the informed consent procedure, participants agreed to participate in a baseline interview, monthly follow-up interviews, and brief surveys after overdose incidents. Project staff installed the app on the participant’s smartphone and provided app training, which included watching an animated training video explaining app use and practicing using the app to send and receive alerts with project staff. Naloxone training included recognizing the signs of opioid overdose, practicing rescue breathing on a CPR dummy, and demonstrating how to administer intranasal naloxone. All participants received a kit containing 2 doses of intranasal naloxone. The UnityPhilly app enabled them to report opioid overdose events and to receive notifications about opioid overdose events reported by other members in their proximity. Participants received US $25 in cash for the baseline interview and US $5 for each completed follow-up monthly interview or incident survey. No compensation was offered or given for the use of the app to signal or respond to overdose incidents. More details about the study are available in prior publications [34].

**Figure 2:**
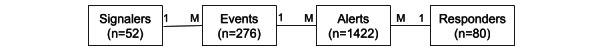
Entities in the UnityPhilly data set. M: many.

The data used for this analysis consist of 4 components (Textbox 1 and Figure 2).

**Figure 3:**
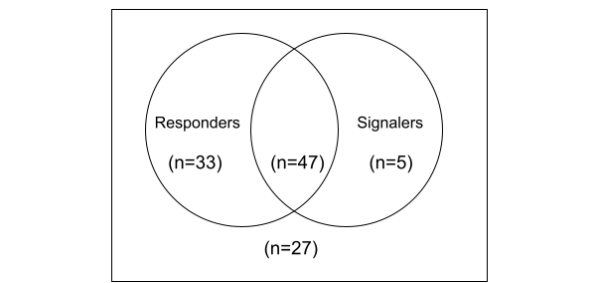
Distribution of responders and signalers in the UnityPhilly data set.

Of the 112 volunteers recruited to UnityPhilly, 27 (24.1%) were completely inactive as either signaler or responder (ie, they did not send or respond to a single alert). Of the remaining 85 volunteers, 80 (94%) received at least 1 alert and were defined as *responders*, and 52 (61%) who signaled at least 1 event were defined as *signalers* (many volunteers served in both roles). Figure 3 presents the distribution of responders and signalers.

**Figure 4:**
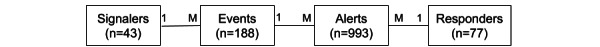
Sample used for this study. M: many.

Events that were canceled by the signaler for any reason were considered false alarms. For this analysis, we excluded these events because we were not able to distinguish between alarms ignored by the responder and alarms that were canceled before the responder had a chance to respond. Figure 4 describes the sample.

We used *alerts* as a unit of analysis.

**The 4 components of the data used for analysis.**

**Event**

- This refers to an opioid overdose event. An event’s characteristics are true or false alarm, signaler, weekday or weekend, and day or night.

**Signaler**

- This refers to a UnityPhilly user who witnesses an event and reports it to the system using the UnityPhilly app. A signaler’s characteristics are age, gender, housing status, employment status, naloxone carriage adherence before joining the UnityPhilly community, opioid overdose witnessing experience before joining the UnityPhilly community, and experience in administering naloxone to a person experiencing an overdose before joining the UnityPhilly community.

**Responder**

- This refers to a UnityPhilly member who is selected by the UnityPhilly system (based on their location and estimated time of arrival) and notified in their UnityPhilly app about an event. The responder’s characteristics are the same as those of the signaler.

**Alert**

- This refers to a notification sent to a specific responder about a specific event. The UnityPhilly app enables the responder to accept or decline an alert. However, many alerts are ignored, that is, neither accepted nor declined. An alert’s characteristics are distance between the potential responder and the event scene at the moment of the alert, the number of previous alerts received by the responder since joining UnityPhilly, the number of previous false alerts received by the responder since joining, the number of previous alerts received by the responder since joining that were initiated by the same signaler, the number of previous false alerts received by the responder since joining that were initiated by the same signaler, the number of previous responses by the responder since joining, the number of previous responses to false alerts received by the responder since joining, and the number of previous responses to false alerts initiated by the same signaler that were received by the responder since joining.

### Analytical Approach

**Figure 5:**
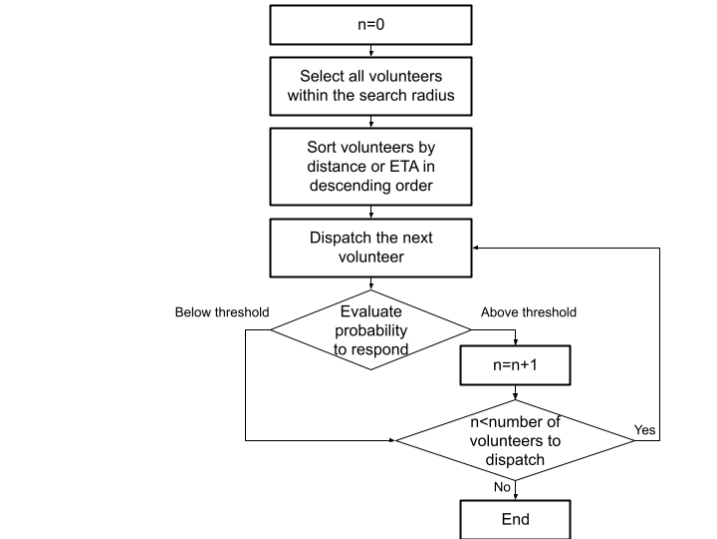
Integration of the probability to respond into the dispatch algorithm. ETA: estimated time of arrival.

We used multiple analytical methods to classify the behavior of each volunteer identified as being in the proximity of an overdose event. We integrated data on specific volunteers and events into the dispatch algorithm in such a way that for each dispatched volunteer who is most likely to ignore the alert, an additional volunteer is dispatched right away (if available), until the maximum number of volunteers to be dispatched is reached, or no more volunteers are available. Volunteers for whom the algorithm predicts a low probability of response are still dispatched and thus are given the chance to respond. Figure 5 depicts this process.

We tested 4 models, based on different configurations of variables, to predict whether a given responder is likely to respond to a given event (Textbox 2 and Table 1).

**The 4 models tested in this study.**

**Model 1**

- This model is based solely on historic events and alerts data, incorporating no other data related to the potential responders.

**Model 2**

- This model is based on the events and alerts data, but it also integrates data on the responders’ patterns of behavior through their previous experience in the volunteer first responder network, including previous alerts and false alerts, and previous responses, including responses to false alerts.

**Model 3**

- This model is based on the events and alerts data, as well as respondents’ personal and demographic data, and ignores their previous experience in the network.

**Model 4**

- This model is based on the events and alerts data, as well as respondents’ personal and demographic data, and dynamically calculates the
  frequent responder
  indicator that represents the responder’s experience in the community before a specific alert. This indicator was calculated as follows:
  <6 alerts: no
  6-10 alerts and response rate ≥50%: yes
  11-20 alerts and response rate ≥40%: yes
  21-30 alerts and response rate ≥30%: yes
  ≥31 alerts and response rate ≥25%: yes
  Otherwise: no

**Table 1:** Data used in each model.

Data	Model			
	1	2	3	4

Events and alerts data (weekday or weekend, day or night, and distance [m])	✓	✓	✓	✓
Responder’s previous experience in UnityPhilly (previous alerts, previous false alerts, previous alerts by the same signaler, previous false alerts by the same signaler, previous responses, previous responses to false alerts, and previous responses to false alerts by the same signaler)		✓		✓
Responders’ demographic data (age, gender, housing status, and employment status)			✓	✓
Responders’ condition-specific characteristics (naloxone carriage adherence, history of witnessing opioid overdoses before joining UnityPhilly, and history of administering naloxone before joining UnityPhilly)			✓	✓
Frequent responder indicator (recalculated after each alarm)				✓

### Classification

The classification analysis for all models was conducted using four classification algorithms suitable for binary classification: (1) the J48 decision tree algorithm, which is an extension of the C4.5 algorithm, implemented in Weka software (University of Waikato) used in the research; (2) random forest; (3) neural network (multilayer perceptron); and (4) logistic regression. The J48 algorithm creates univariate decision trees for classification and provides effective alternatives to other classification methods. The choice of the *best* classification model is based on the combination of different evaluation metrics. The main interest was to identify the model that succeeds in correctly classifying *any answer* class.

We used 4 evaluation metrics: accuracy, *F*-score, precision, and recall. Accuracy is the overall percentage of correctly classified instances. The *F*-score is the harmonic mean of the recall and precision metrics and can take values ranging between 0 (none of the instances were correctly classified) and 1 (all instances were correctly classified). Precision is the percentage of true positively classified instances out of all positively classified instances. Recall is the percentage of positively classified instances out of all positive instances. The best way to explain the trends found in this analysis is to explain the differences in the recall metric among the different classification algorithms and among the different classes.

Because of the relatively small overall number of cases in the data set, we did not use a percentage split for the training and test sets for models 1 to 3; instead, we used a cross-validation option with 10 folds. Model 4 includes the additional synthetic dichotomous variable called *frequent responder* that reflects the previous behavior of the responder. The variable is dynamically updated; therefore, a responder can change their behavior several times throughout the research period—from being active to inactive or vice versa. As the *frequent responder* variable cannot be treated as an independent sequence of values and behavioral patterns that must be preserved, there is no option to use cross-validation for classification analysis. For this reason, we split the data set into a training set with 66.9% (664/993) of the data and a test set with 33.1% (329/993) of the data.

All 4 algorithms were used for a binary classification task in a baseline analysis that included only the events and alerts data (model 1 in Table 1). The obtained results provide the baseline for comparison with the additional data related to the responder’s previous experience data (models 2, 3, and 4 in Table 1). We claim that building a model that considers the responders’ behavioral characteristics can improve the use of the dispatch algorithm. In this kind of analysis, precision in predicting nonresponse is more important than precision in predicting response because in the former case a mistake will delay the dispatch of a substitute responder, whereas in the latter case a mistake will result in the dispatch of too many volunteers.

The comparison between all classification techniques and all evaluation metrics for the 4 models is presented in Multimedia Appendix 1.

### Ethical Considerations

All study procedures were approved by the Drexel University Institutional Review Board and registered with ClinicalTrials.gov (NCT03305497). Study enrollment included written informed consent. All data used for this research were deidentified. Participants received US $25 in cash for the baseline interview and US $5 for each completed follow-up monthly interview or incident survey. No compensation was offered or given for use of the app to signal or respond to overdose incidents.

## Results

The results of this study are derived from an analysis of emergency events, volunteer participants’ demographics, and behavior patterns.

### Description of the Sample

Table 2 presents the characteristics of overdose events.

Table 3 presents the distribution and correlation of the responders’ characteristics. Cramér *V* was used for categorical variables, and Spearman ρ was used for ordinal variables. ANOVA tests for age differences among the different subgroups of categorical or ordinal variables did not reveal any significant differences at the 5% significance level.

**Table 2:** Description of overdose event characteristics (n=188).

Variables			Values
**Weekdays and weekends, n (%)^a^**			
	Weekday	136 (72.3)	
	Weekend	52 (27.7)	
**Days and nights, n (%)^a^**			
	Day	133 (70.7)	
	Night	55 (29.3)	
Distance (meters; n=162^b^), mean (SD); median (IQR)^c^			3326 (2784); 2595 (955.09-5567.75)

^a^Cramér correlation between weekday/weekend and day/night is 0.006.

^b^For 26 (13.8%) of the 188 overdose events, distance data were not available.

^c^Distance during weekdays: mean 3611 (SD 2871) meters; distance during weekends: mean 2537 (SD 2384) meters; *P*=.03; distance during the day: mean 3507 (SD 2724) meters; distance during the night: mean 2870 (2910) meters; *P*=.19.

**Table 3:** Distribution and correlation of responders’ characteristics (n=80).

Variable			Values, n (%)	Gender	Naloxone carriage adherence	Homelessness	Employment	History of witnessing an opioid overdose	History of administering naloxone	Age
**Age^a^**										
		*r*	—^b^	0.07	0.18	0.13	0.14	−0.08	−0.07	1
		*P* value	—	.54	.12	.26	.22	0.52	.54	—
**Gender**										
	r		—	1	0.25	0.42	0.18	0.19	0.14	0.07
	*P* value		—	—	.27	<.001	.25	.35	.58	.54
	Male		35 (44)	—	—	—	—	—	—	—
	Female		44 (55)	—	—	—	—	—	—	—
	Intersex		1 (1)	—	—	—	—	—	—	—
**Naloxone carriage adherence**										
	r		—	0.25	1	0.37	0.27	−0.18	−0.21	0.18
	*P* value		—	.27	—	.02	.16	.05	.05	.12
	All the time		36 (45)	—	—	—	—	—	—	—
	Often		22 (28)	—	—	—	—	—	—	—
	Sometimes		10 (13)	—	—	—	—	—	—	—
	Seldom		2 (3)	—	—	—	—	—	—	—
	Never		10 (13)	—	—	—	—	—	—	—
**Homelessness**										
	r		—	0.42	0.37	1	0.37	0.22	0.09	0.13
	*P* value		—	<.001	.02	—	.004	.16	.46	.26
	Homeless		22 (28)	—	—	—	—	—	—	—
	Not homeless		58 (73)	—	—	—	—	—	—	—
**Employment**										
	r		—	0.18	0.27	0.37	1	0.15	0.14	0.14
	*P* value		—	.25	.16	.004	—	.16	.58	.22
	Part time		11 (14)	—	—	—	—	—	—	—
	Full time		18 (23)	—	—	—	—	—	—	—
	Unemployed		51 (64)	—	—	—	—	—	—	—
**History of witnessing an opioid overdose (number of times)**										
	r		—	0.19	−0.18	0.22	0.15	1	0.63	−0.08
	*P* value		—	.35	.05	.16	.16	—	<.001	.52
	≤20		48 (60)	—	—	—	—	—	—	—
	21-40		20 (25)	—	—	—	—	—	—	—
	>40		12 (15)	—	—	—	—	—	—	—
**History of administering naloxone (number of times)**										
	r		—	0.14	−0.21	0.09	0.14	0.63	1	−0.07
	*P* value		—	.58	.05	.46	.58	<.001	—	.54
	≤20		61 (81)	—	—	—	—	—	—	—
	21-40		7 (9)	—	—	—	—	—	—	—
	>40		7 (9)	—	—	—	—	—	—	—

^a^Age (y): mean 40.31 (SD 10.41); median 39.5 (IQR 32-47.75).

^b^Not applicable.

Significant correlations were found between gender and homelessness (*P*<.001) as well as between history of witnessing an opioid overdose and history of administering naloxone (*P*<.001).

### Response Patterns

**Figure 6:**
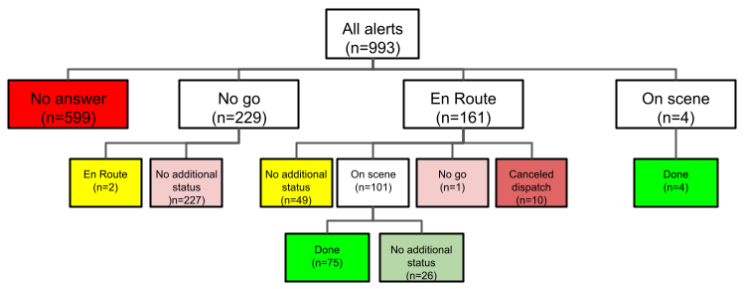
Response patterns (refer to Textbox 3 for an explanation of the terms used in this figure).

Textbox 3 and Figure 6 present how the alerted volunteers responded (true alarms only; n=993). Responders could change their decision.

**Volunteers’ response patterns.**

**No answer**

- Responder ignored the alert. This was the final status in 60.3% (599/993) of the alerts.

**No go**

- Responder notified the system that they are not able to respond. This was the final status in 23% (228/993) of the alerts.

**En route**

- Responder notified the system that they are on the way to the scene. This was the final status in 5.1% (51/993) of the alerts.

**On scene**

- Responder notified the system that they are on the scene. This status can be set automatically by the system (based on the responder’s location) or manually by the responder. This was the final status in 2.6% (26/993) of the alerts.

**Done**

- Responder performed the treatment. This was the final status in 7.9% (79/993) of the alerts.

**Canceled dispatch**

- This was the final status in 1% (10/993) of the alerts.

### Classification Analysis of Response Patterns

**Figure 7:**
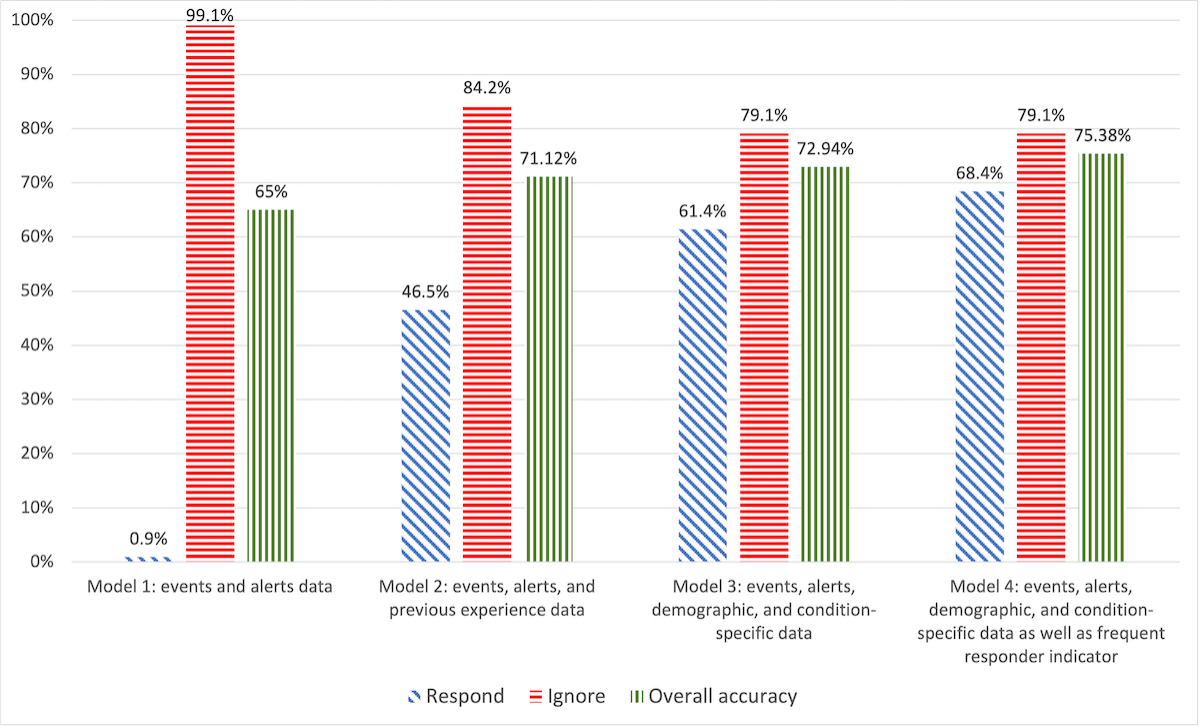
Classification accuracy of models 1 to 4 using the test set (n=329).

Figure 7 presents the ability of each model to predict the responder’s behavior. To compare model 4 with the other models, all models were tested using the test set of alerts (n=329).

For the test set, model 4 provided the best classification accuracy both overall and for ignored alerts. Model 3 provided the same classification accuracy for ignored alerts, slightly lower accuracy overall, and lower accuracy for answered alerts. Model 2 provided the best classification accuracy for ignored alerts; however, its accuracy was lower overall and significantly lower for answered alerts. Model 1’s classification accuracy was the lowest.

**Figure 8:**
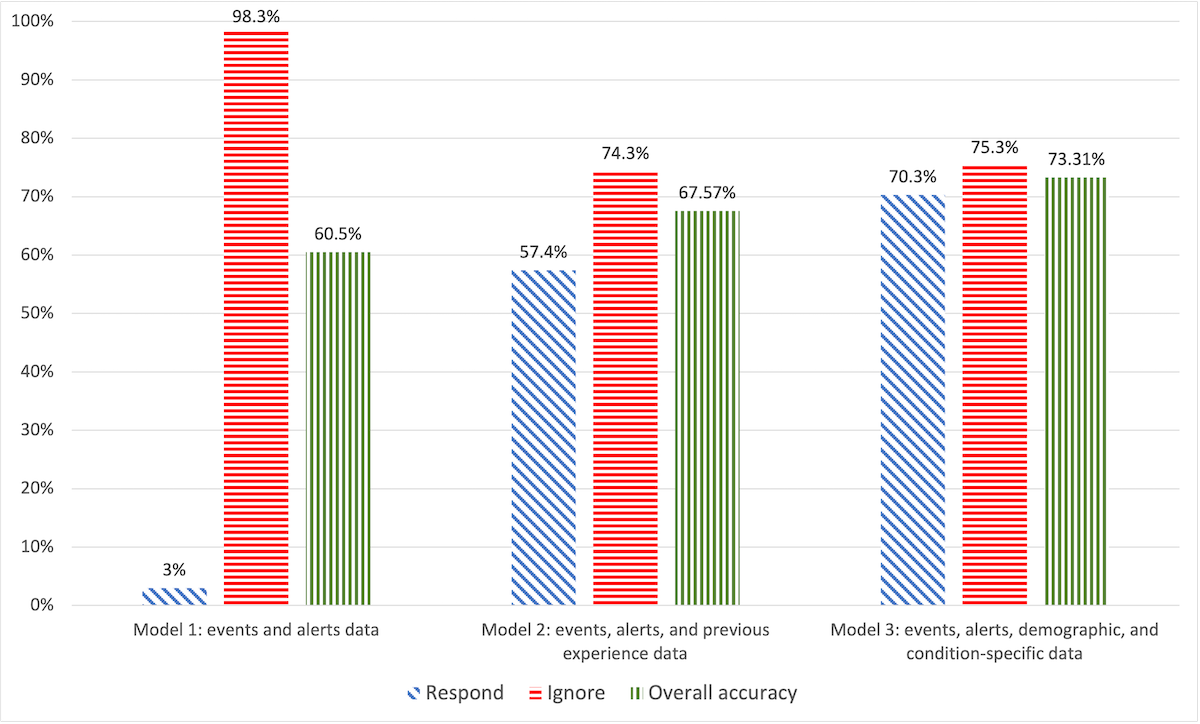
Classification accuracy of models 1 to 3 using the full set (n=993).

Figure 8 presents the ability of models 1 to 3 to classify the responder’s behavior, using the full data set (n=993).

For the full set, model 3 provided the best classification accuracy. Model 2 had similar accuracy for ignored events and lower accuracy both overall and for answered events. Model 1’s classification accuracy was the lowest. Model 4 was not tested with the full set because the construction of the *frequent responder* variable requires training.

**Figure 9:**
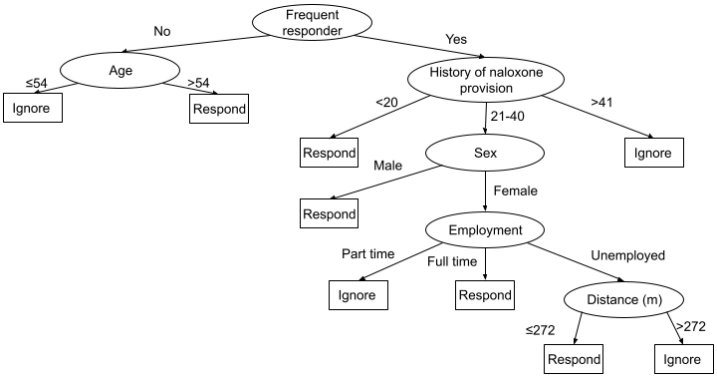
J48 decision tree for model 4 for the test set.

Figure 9 presents the J48 decision tree for model 4 for the test set.

The analysis of the classification tree reveals 5 possible routes to the *response* result: infrequent responders aged >54 years, frequent responders who administered naloxone <20 times, male frequent responders who administered naloxone 21 to 40 times, fully employed female frequent responders who administered naloxone 21 to 40 times, and unemployed female frequent responders who administered naloxone 21 to 40 times in situations where the distance to the scene was <272 meters.

We have to remember that the overall accuracy is not very high and that there are false-positive and false-negative statistical errors in the classification output. A false-positive error occurs when the ignored alert is classified as a responded alert, and a false-negative error occurs when the responded alert is classified as an ignored alert.

### Potential Time Savings

**Figure 10:**
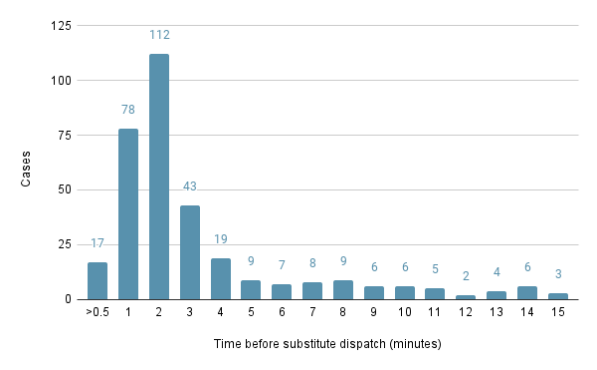
Time before substitute dispatch (n=334).

Substitute responders (responders who were not initially selected by the algorithm) were used in 73.4% (138/188) of the events. Substitute responders received 33.6% (334/993) of the alerts. Figure 10 presents the lengths of the delays (in min) before substitute responders were dispatched.

### Factors Affecting Willingness to Respond to an Opioid Overdose Event

Table 4 presents the analysis of differences between alerts that were ignored and alerts that resulted in some responses (*en route*, *no go*, or *on scene*).

Significant differences between responded alerts and ignored alerts were found for the following variables: gender (higher response rate by male volunteers; *P*=.05), naloxone carriage adherence (*P*<.001), employment (higher response rate by volunteers who were unemployed; *P*<.001), age (slightly higher average age among volunteers who responded; *P*=.003), the number of previous alerts (higher among volunteers who responded; *P*=.003), previous false alerts (higher among volunteers who responded; *P*=.003), previous false alerts by the same signaler (lower among volunteers who responded; *P*=.02), previous responses (higher among volunteers who responded; *P*<.001), and previous responses to false alerts (higher among volunteers who responded; *P*<.001).

**Table 4:** Differences between responded alerts and ignored alerts (n=993).

Variable		Responded alerts (n=394)	Ignored alerts (n=599)	*P* value	
**Weekdays and weekends, n (%)**					.49^a^
	Weekday	289 (73.4)	451 (75.3)		
	Weekend	105 (26.6)	148 (24.7)		
**Days and nights, n (%)**					.44^a^
	Day	282 (71.6)	415 (69.3)		
	Night	112 (28.4)	184 (30.7)		
**Sex, n (%)**					.05^a^
	Male	182 (46.2)	239 (39.9)		
	Female	212 (53.8)	360 (60.1)		
**Naloxone carriage adherence, n (%)**					<.001^a,b^
	All the time	115 (29.2)	241 (40.2)		
	Most of the time	174 (44.2)	150 (25)		
	Sometimes	29 (7.4)	59 (9.8)		
	Seldom	4 (1)	17 (2.8)		
	Never	72 (18.3)	132 (22)		
**Homelessness, n (%)**					.18^a^
	Yes	68 (17.3)	124 (20.7)		
	No	326 (82.7)	475 (79.3)		
**Employment, n (%)**					<.001^a^
	Part time	18 (4.6)	70 (11.7)		
	Full time	70 (17.8)	110 (18.4)		
	Unemployed	306 (77.7)	419 (69.9)		
Age (y), mean (SD)		42.91 (11.86)	40.47 (13.11)	.003^c^	
Previous alerts, mean (SD)		25.00 (20.96)	21.09 (20.31)	.003^c^	
Previous alerts by the same signaler, mean (SD)		3.35 (5.12)	3.52 (5.58)	.63^c^	
Previous false alerts, mean (SD)		7.70 (7.00)	6.39 (6.46)	.003^c^	
Previous false alerts by the same signaler, mean (SD)		0.70 (1.35)	0.96 (2.03)	.018^c^	
Previous responses, mean (SD)		14.27 (14.01)	6.74 (10.40)	<.001^c^	
Previous responses to false alerts, mean (SD)		1.39 (1.85)	0.82 (1.59)	<.001^c^	
Previous false alerts by the same signaler, mean (SD)		0.13 (0.42)	0.13 (0.50)	.89^c^	
Distance, mean (SD)		1947.24 (2290.24)	1726.98 (2127.02)	.16^c^	
**History of witnessed overdoses (number of times), n (%)**					.54^a^
	≤20	247 (62.7)	382 (63.8)		
	21-40	80 (20.3)	106 (17.7)		
	>40	67 (17)	111 (18.5)		
**History of naloxone administration (number of times), n (%)**					.07^a^
	≤20	299 (86.4)	453 (80.6)		
	21-40	14 (4)	38 (6.8)		
	>40	33 (9.5)	71 (12.6)		

^a^*P* value for the chi-square test for the test of independence.

^b^*P* value for the Kendall τ test for ordinal variables.

^c^*P* value for the 2-tailed independent samples *t* test.

## Discussion

### Principal Findings

To the best of our knowledge, this is the first study that integrates the predictions of VFRs’ response behavior into dispatch algorithms. We found that volunteers’ past response behavior is the most influential predictor of future response behavior. Our findings suggest that the behavior-based approach can be applied to VFR dispatch to achieve a better response rate of the network as a whole.

### Profiling of Responders and Personalization of VFR Dispatch

Model 1 used events and alerts data only and completely ignored any volunteer-related data. The ability of this model to predict volunteers’ behavior was extremely low—only approximately 0.9% (3/329) of the responded alerts were classified correctly. This model would lead to the dispatch of all available volunteers, resulting in burnout, a “diffusion of responsibility,” and low willingness to respond. We conclude that this model is unacceptable.

Model 2 assumed that the volunteers are completely anonymous and that the algorithm knows only their previous behavior in the VFR network. Using these data as well as events and alerts data, model 2 correctly predicted 84.2% (277/329) of the cases in the test set in which responders ignored an alert. Lower prediction accuracy for the full data set (738/993, 74.3%) can be explained by the inclusion of early events for which the model did not have enough data about previous behavior. The ability of model 2 to predict that a volunteer will respond to an event was lower (153/329, 46.5% for the test set and 570/993, 57.4% for the full data set). On the one hand, this model is expected to improve the response rate of the network, but, on the other hand, in half of the cases in which a volunteer responds, another volunteer would be dispatched unnecessarily. We conclude that this model should be used only if the responders are completely anonymous.

Model 3 used events and alerts data, responders’ demographics, their prior experience of witnessing an opioid overdose, their prior experience in administering naloxone, and their naloxone carriage adherence *before* joining the VFR network. This model’s ability to predict that a volunteer will ignore an alert was similar to that of model 2, but its ability to predict that a volunteer will respond to an event was higher (202/329, 61.4% in the test set and 698/993, 70.3% in the full data set). A closer look at the decision tree of this model reveals that the most influential variables were related to the volunteer’s experience before joining the network: naloxone carriage adherence and the provision of naloxone to those experiencing an overdose. We conclude that this model can be used if data about a volunteer’s app use behavior in the network are not currently available (eg, during the period between recruiting the volunteer and until they receive enough alerts).

Model 4 used all available data, including the frequency of events and alerts, volunteers’ demographics, their prior overdose witnessing and naloxone provision experience before joining the VFR network, and their response behavior in the VFR network (according to the *frequent responder* indicator recalculated after each event). The ability of this model to predict that a volunteer will ignore an alert was similar to that of model 3, but its ability to predict that a volunteer will respond to an event was higher (225/329, 68.4% in the test set). The decision tree presented in Figure 9 reveals that the *frequent responder* indicator was the most influential variable. We conclude that model 4 achieves the best prediction accuracy and should be preferred whenever the necessary data are available.

### Generalizability of the Proposed Approach

The dispatch of substitute responders is relevant whenever the initial subset of closest volunteers do not provide a response and is a common approach in VFR systems. However, valuable time is lost until nonresponse is identified. Although existing algorithms are based on technical variables such as distance from the scene and the ETA, this study introduces a completely new variable: volunteers’ behavior and their probability to respond to a specific alert. The demonstrated importance of considering multiple factors in volunteer demographics and behavioral characteristics and the insights from the models we have tested are applicable wherever volunteer dispatch optimization is important. Such challenges are found in areas as diverse as food rescue operations [53], OHCA response [54,56], and mass casualty events [57]. Following our approach, these domains and more may find value in testing different sets of demographic and behavioral factors. Predicting the response behavior of candidate responders in advance of dispatch can allow any VFR system to choose the best possible response candidates based not only on ETA or location but also on the probability of actual response. The potential time savings depend on the network-specific period of time until a nonresponsive volunteer is considered unavailable, and a substitute responder is dispatched. The longer this time period, the greater the potential savings provided by a predictive dispatch algorithm.

The data used in our algorithm can be divided into four categories (Table 1): (1) event characteristics, (2) past responder behavior, (3) demographics, and (4) certain parameters specific to a medical condition relevant to the VFR network. The first 3 categories are directly generalizable because most responder mobilization apps collect and store these data, which, based on our findings, can be harvested for improved dispatch algorithms. The fourth data category includes factors that may differ depending on the medical condition relevant to the VFR network.

### Factors Affecting Volunteers’ Decision to Respond

Herein, we provide a brief discussion of the factors that affect volunteers’ decisions to respond. A full analysis of these factors is beyond the scope of this research and should be pursued using a larger sample that may provide generalizability.

Experience, including the experience gained both before and after joining the VFR community, was found to be the most influential factor in volunteers’ willingness to respond. In model 3, naloxone carriage adherence and experience in the provision of naloxone were the most influential factors, whereas in model 4, the *frequent responder* indicator was the most influential factor. Significant differences were found between responded alerts and ignored alerts for the following variables: naloxone carriage adherence, previous alerts, previous false alerts, previous false alerts by the same signaler, previous responses, and previous responses to false alerts.

Part-time employment led to lower willingness to respond.

The average age of volunteers who responded to alerts was a little higher than the average age of volunteers who ignored alerts. Model 4 revealed that age is an important factor for volunteers who are not *frequent responders*: volunteers aged >54 years are expected to respond.

Male volunteers had a higher willingness to respond, but this difference had borderline significance (*P*=.05). The results of model 4 were consistent with this difference.

### Comparison With Prior Work

VFR dispatch has evolved in the last decades, thanks to technological advances and a better understanding of VFR network management. In a pretechnology era, VFRs (eg, volunteer firefighters) were alerted by sirens or other means rather than individually dispatched. Once pagers and SMS text messaging technology became available, the first generation of individual dispatch based on usual location was implemented (eg, the study by Zijlstra et al [43]). Further technological advances, including smartphone apps and GPS, enabled the second generation of individual dispatch based on current location [34,36,46,47] and the integration of VFRs’ feedback into the algorithm [34]. The integration of the probability to respond based on event characteristics as well as VFRs’ demographic data and previous behavior into the dispatch algorithm constitutes the third generation of individual dispatch, making this one of the first studies to harness the power of predictive analytics for VFR dispatch.

### Limitations

A relatively small sample for a specific condition (opioid overdose) and a specific emergency intervention (the provision of naloxone) was used. The sample has specific socioeconomic characteristics: it included a significant proportion of people experiencing homelessness and those who were unemployed (volunteers may have lower motivation to help owing to these destabilizing factors), as well as a significant proportion of people dependent on drugs (volunteers may have lower response rates when intoxicated). The setting was very specific: a large number of outdoor opioid overdoses within a relatively small geographic area. The responders were aware that there were many trained bystanders nearby, and this could have led to the “diffusion of responsibility” phenomenon and reduced the willingness to respond. No randomization or control group was used.

### Future Research

The proposed approach should be tested with a larger sample and for different conditions and interventions. A randomized study comparing the outcomes of the proposed dispatch algorithm with those of a regular location-based dispatch algorithm should be considered.

Machine learning techniques should be considered to calculate the *frequent responder* indicator. Future studies should examine whether the probability that a specific responder will respond to a specific event can be used instead of a binary indicator.

Further research is necessary on whether the proposed approach may have implications for multisided networks dispatching nonemergency services, such as ride sharing and package delivery.

### Conclusions

In this research, we proposed a way to improve dispatch algorithms in VFR networks based on the individual characteristics of the volunteers and their behavior. We have shown that even in a relatively small sample, a classification model can predict with fair accuracy whether a specific volunteer will respond to a specific event or ignore it. Such prediction may improve the dispatchers’ decision-making process and enable the dispatch of substitute responders without delay.

Our findings can help VFR network administrators in their continual efforts to improve the response rates and response times of their networks and to save lives.

## Appendix

Multimedia Appendix 1 Comparison of different algorithms for models 1 to 4.

## Abbreviations

AED: automatic external defibrillator
CPR: cardiopulmonary resuscitation
EMS: emergency medical services
ERC: emergency response community
ETA: estimated time of arrival
OHCA: out-of-hospital cardiac arrest
VFR: volunteer first responder

## Data Availability

The data sets analyzed during this study are not publicly available owing to Health Insurance Portability and Accountability Act (HIPAA) guidelines regarding patient privacy and the sensitivity of raw location data. Requests for data can be sent to the corresponding author.
