# Supplementary material for: Predictive Dispatch of Volunteer First Responders: Algorithm Development and Validation
Source: JMIR Mhealth Uhealth. 2023 Nov 28;11:e41551. doi: 10.2196/41551 (PMC10716760; doi:10.2196/41551)
Supplement: Multimedia Appendix 1 [file mhealth_v11i1e41551_app1.docx]

Multimedia Appendix 1. Comparison of different algorithms for models 1 to 4

| Classification algorithm🡪 | **J 48**** | | | | **Random Forest** | | | | **Neural Network**  **Multilayer Perceptron** | | | | **Logistic Regression** | | | |
| --- | --- | --- | --- | --- | --- | --- | --- | --- | --- | --- | --- | --- | --- | --- | --- | --- |
| Accuracy measure 🡪 | Accuracy | Precision | Recall | F | Accuracy | Precision | Recall | F | Accuracy | Precision | Recall | F | Accuracy | Precision | Recall | F |
| **Model 1** | 60.52% | Class 0=  0.61  Class1=  0.55 | Class 0=  0.98  Class1=  0.03 | 0.48 | 63.34% | Class0=  0.69  Class1=  0.54 | Class 0=  0.72  Class1=  0.5 | 0.63 | 59.1% | Class 0=  0.63  Class1=  0.48 | Class 0=  0.78  Class1=  0.3 | 0.57 | 60.22% | Class0=  0.6  Class1=  0.46 | Class 0=  0.99  Class1=  0.01 | 0.46 |
| **Model 2** | 67.27% | Class 0=  0.72  Class1=  0.59 | Class 0=  0.75  Class1=  0.56 | 0.67 | 65.56% | Class 0=  0.69  Class1=  0.58 | Class 0=  0.77  Class1=  0.48 | 0.65 | 70.39% | Class 0=  0.72  Class1=  0.66 | Class 0=  0.83  Class1=  0.52 | 0.7 | 67.57% | Class 0=  0.68  Class1=  0.66 | Class 0=  0.88  Class1=  0.37 | 0.65 |
| **Model 3*** | 72.41% | Class 0=  0.79  Class1=  0.64 | Class 0=  0.75  Class1=  0.69 | 0.73 | 69.49% | Class 0=  0.74  Class1=  0.62 | Class 0=  0.75  Class1=  0.61 | 0.69 | 70.39% | Class 0=  0.75  Class1=  0.63 | Class 0=  0.77  Class1=  0.6 | 0.7 | 64.25% | Class 0=  0.67  Class1=  0.58 | Class 0=  0.82  Class1=  0.38 | 0.62 |
| **Model 4** | 69.29% | Class 0=  0.75  Class1=  0.61 | Class 0=  0.73  Class1=  0.64 | 0.69 | 70.29% | Class 0=  0.75  Class1=  0.63 | Class 0=  0.77  Class1=  0.6 | 0.7 | 69.69% | Class 0=  0.74  Class1=  0.62 | Class 0=  0.76  Class1=  0.6 | 0.7 | 68.98% | Class 0=  0.72  Class1=  0.63 | Class 0=  0.79  Class1=  0.54 | 0.69 |

Class 0 = no response

Class 1 = any answer

* best model according to overall accuracy, highest level of precision value for class=1 (any answer) and highest level of recall value for class=1 (any answer)

** best algorithm according to overall accuracy, highest level of precision value for class=1 (any answer) and highest level of recall value for class=1 (any answer)
